# Hybrid model for precise hepatitis-C classification using improved random forest and SVM method

**DOI:** 10.1038/s41598-023-36605-3

**Published:** 2023-08-01

**Authors:** Umesh Kumar Lilhore, Poongodi Manoharan, Jasminder Kaur Sandhu, Sarita Simaiya, Surjeet Dalal, Abdullah M. Baqasah, Majed Alsafyani, Roobaea Alroobaea, Ismail Keshta, Kaamran Raahemifar

**Affiliations:** 1 Department of Computer Science and Engineering, Chandigarh University, Gharuan, Mohali, Punjab 140413 India; 2 College of Science and Engineering, Qatar Foundation, Hamad Bin Khalifa University, Doha, Qatar; 3 Apex Institute of Technology (CSE), Chandigarh University, Gharuan, Mohali, Punjab 140413 India; 4 Amity School of Engineering and Technology, Amity University Haryana, Gurugram, India; 5 Department of Information Technology, College of Computers and Information Technology, Taif University, Taif, 21974 Saudi Arabia; 6 Department of Computer Science, College of Computers and Information Technology, Taif University, P. O. Box 11099, Taif, 21944 Saudi Arabia; 7 Computer Science and Information Systems Department, College of Applied Sciences, AlMaarefa University, Riyadh, Saudi Arabia; 8 College of Information Sciences and Technology, Data Science and Artificial Intelligence Program, Penn State University, State College, PA 16801 USA; 9 School of Optometry and Vision Science, Faculty of Science, University of Waterloo, 200 University, Waterloo, ON N2L3G1 Canada; 10 Faculty of Engineering, University of Waterloo, 200 University Ave W, Waterloo, Canada

**Keywords:** Biotechnology, Medical research, Computational biology and bioinformatics, Computational models, Computational neuroscience

## Abstract

Hepatitis C Virus (HCV) is a viral infection that causes liver inflammation. Annually, approximately 3.4 million cases of HCV are reported worldwide. A diagnosis of HCV in earlier stages helps to save lives. In the HCV review, the authors used a single ML-based prediction model in the current research, which encounters several issues, i.e., poor accuracy, data imbalance, and overfitting. This research proposed a Hybrid Predictive Model (HPM) based on an improved random forest and support vector machine to overcome existing research limitations. The proposed model improves a random forest method by adding a bootstrapping approach. The existing RF method is enhanced by adding a bootstrapping process, which helps eliminate the tree’s minor features iteratively to build a strong forest. It improves the performance of the HPM model. The proposed HPM model utilizes a ‘Ranker method’ to rank the dataset features and applies an IRF with SVM, selecting higher-ranked feature elements to build the prediction model. This research uses the online HCV dataset from UCI to measure the proposed model’s performance. The dataset is highly imbalanced; to deal with this issue, we utilized the synthetic minority over-sampling technique (SMOTE). This research performs two experiments. The first experiment is based on data splitting methods, K-fold cross-validation, and training: testing-based splitting. The proposed method achieved an accuracy of 95.89% for k = 5 and 96.29% for k = 10; for the training and testing-based split, the proposed method achieved 91.24% for 80:20 and 92.39% for 70:30, which is the best compared to the existing SVM, MARS, RF, DT, and BGLM methods. In experiment 2, the analysis is performed using feature selection (with SMOTE and without SMOTE). The proposed method achieves an accuracy of 41.541% without SMOTE and 96.82% with SMOTE-based feature selection, which is better than existing ML methods. The experimental results prove the importance of feature selection to achieve higher accuracy in HCV research.

## Introduction

Healthcare data analysis is a complex and critical task that requires high skill to predict the disease type and its cure. Manual healthcare-based data analysis takes high time, and accuracy is also a significant challenge, which motivates the researchers to develop an automatic system to predict the disease type accurately and suggest a cure^1^. Hepatitis is one of the most common diseases worldwide, caused by infection via blood. Once a patient tests positive for HCV needs immediate attention. Early and accurate detection helps to save a patient life^2^. HCV affects liver functionality. The liver is the most significant organ in the human body, performing more than five hundred plus essential tasks. Hepatitis is one of the severe diseases that affect liver functionality.

As a result, the liver can suffer inflammatory conditions. An infection of a virus usually causes Hepatitis. However, there are other potential causes, i.e., effects of toxins, medications, drugs, and liquor^3^. According to a World Health Organization survey, Hepatitis has a higher mortality rate worldwide than other chronic diseases. Hepatitis disease can be divided into several categories, i.e., Hepatitis-A to Hepatitis-E. Hepatitis C is the most severe and deadly disease, but early detection can helps recover without losing any liver damage. The initial stage of Hepatitis C is termed acid hepatitis; after five months, it becomes a critical disease and leads to long sickness. It directly strikes the internal organs, i.e., the liver and stomach. The body’s defense function releases inflammatory hormones as a direct consequence.

According to a World Health Organization survey, Hepatitis has a higher mortality rate worldwide than other chronic diseases. Hepatitis disease can be divided into several categories, i.e., Hepatitis-A to Hepatitis-E. Hepatitis C is the most severe and deadly disease, but early detection can helps recover without losing any liver damage. The initial stage of Hepatitis C is termed acid hepatitis; after 5 months, it becomes a critical disease and leads to long sickness. It directly strikes the internal organs, i.e., the liver and stomach. The body’s defense now releases inflammatory hormones^4^.

**Figure 1:**
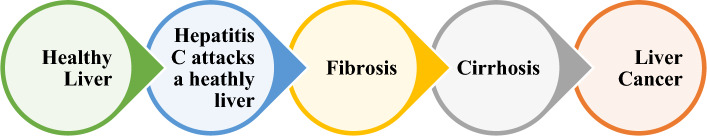
Stages of hepatitis C infection.

Further, chronic Hepatitis-C is an acute disease that does not have a successful vaccine. This disease regularly prompts the origin of severe infections in the body, i.e., liver cirrhosis, fibrosis, and cancer. Figure 1 shows the disease types.

Hepatitis disease has several stages in the body. Liver fibrosis mainly occurs due to any injury mending reaction and tissue damage. Similar cirrhosis is a high-level phase of liver fibrosis with hepatic architecture and vasculature^5^. The risk of liver cancer increases when a proper diagnosis is not taken appropriately. Early detection of Hepatitis via the correct diagnosis of blood samples, known as liver tests and appropriate medicine, can help cure the disease^6^. This liver test includes two primary serum biochemical markers named aminotransferase (ALT) and aspartate aminotransferase (AST)^7^. A patient with a higher level of ALT has more risk of being infected with the hepatitis virus. The patient is recommended for an HCV test. The level of Hepatitis C is detected via the ranks of HCV at 12 weeks. Blood serum markers help predict disease states and reduce medical costs^8^.

The diagnosis process of HCV includes two steps. The first step mainly selects the correct diagnosis parameters, and the second suggests accurately analyzing data^9^. A previous study revealed that ML models help to predict the HCV disease’s stages by incorporating computer-based patient records and clinical decision support. Research^10^ applied different ML techniques for predicting hepatitis C. A prediction model using the artificial neural network (ANN) approach, with gene parameters and the clinical test, is discussed in^11^. Research^11^ utilized ML algorithms to detect the inflammatory severity of hepatitis C and fibrosis stages using serum indices of patients’ data. To predict Hepatitis, research^12^ proposed a prediction model by combining Multilayer Perceptron (MLP) and a genetic algorithm. Research^13^ also applied three ML models, SVM, ANN, and k-Nearest Neighbor (kNN), to predict hepatitis disease. RF is a popular classification algorithm addressing regression and classification problems. It is an appealing candidate for multi-class classification because of its computational efficiency. In addition, its potential to deal with high-dimensional feature data and greater effectiveness under large datasets are crucial strengths over the other ML algorithms.

A diagnosis system using an RF algorithm to classify cirrhosis and hepatitis patients has been developed^14^. ML is a multidisciplinary domain that combines mathematics and computer science to design computer-based algorithms. These algorithms can amplify the predictive accuracy of static laboratory data utilizing probabilistic or analytic models. ML models provide an effective solution for the diagnosis process by detecting and learning different relationships and patterns between clinical data^15^. These models utilize longitudinal information for building the prediction models and can combine the other variables without compromising the risk prediction accuracy. A prediction model based on clinical risk in hepatitis C is challenging because of the non-linear nature of disease progression. This research proposed an HPM for Hepatitis C detection based on IRF and SVM. The key contributions are as follows:

- HPM utilizes a Ranker-based and SMOTE-based feature selection, which helps to select only essential features from the dataset and overcome data Imbalancing. It improves the overall performance of the model.
- This research also overcomes the limitation of the random forest by adding a bootstrapping method in tree construction and next-phase selection. The IRF employs an optimal count of trees. In contrast, conventional RF infers that expanding the count of trees dynamically improves the correctness, which is not feasible in practice. This IRF method helps to eliminate the less critical features from the tree iteratively to build a strong forest, which improves the performance of the RF model.
- We utilized the UCI HCV dataset and performed two experiments to measure the performance of the HPM model. The first experiment is based on the dataset splitting method and k-fold cross-validation. The second experiment is based on feature selection (with SMOTE and without SMOTE).

This research paper is organized as follows: The related work is illustrated in "Existing work". The proposed system is described in "Materials and methods". Experimental results and discussions are represented in "Results and discussion". The concluding remarks and future directions are discussed in "Conclusion and future works".

## Existing work

This section presents the recent work of various researchers’ methods to predict HCV disease. Research^16^ applied different ML techniques for predicting advanced fibrosis using serum biomarkers such as RT, DT, CART, MLR, ADT, GA, REPT, and PSO. The experimental results have proven that ML techniques help predict the liver’s advanced fibrosis due to HCV. Research^17^ used the RF technique to predict Hepatitis C based on lab reports of HIV patients collected from Lucknow hospital in 2019. The experimental results have proven that RF achieves a 98.3% accuracy rate.

Research^18^ proposed a diagnosis system that utilizes an ANN approach to diagnose hepatitis C. The experimental results revealed that the ANN approach correctly diagnoses the disease by achieving 93% accuracy. The proposed method utilizes fibrosis scores and aspartate aminotransferase-to-platelet to develop an automatic diagnosis system to predict the disease. The performance of the diagnostic system is evaluated using the AUC parameter on the HCV dataset of 166 Egyptian children. Research^19^ used the binary LR technique to predict HCV from the laboratory dataset of California University. The proposed model outperforms over existing prediction model by achieving 83% accuracy. The authors suggested that the proposed model produces good accuracy results with less complexity of features to classify the different stages of HCV. Research^20^ proposed a classification model based on ML techniques, i.e., SVM, DT, GB, LR, NB, KNN, XGB, and RF. The proposed system’s performance is measured using sensitivity, type I error, specificity, f-measure, accuracy, type II error rate, and AUROC parameters on datasets of Egyptian patients. The results revealed that kNN achieves the highest accuracy rate of 94.40% over existing ML methods. Research^21^ applied the RF technique to predict hepatitis C from the EHRs of 615 patients. The author suggests that two enzymes, ALT and AST, play an essential role in predicting HCV. The results proved that the ensemble ML method helps doctors predict the patients’ risk of Cirrhosis and HCV more accurately.

Research^22^ used three ML techniques to design a prediction model: SVM, ANN, and KNN. This research calculated two performance measuring parameters, i.e., accuracy and mean square error. A total of 155 clinical cases were used to measure the model performance. MATLAB software is utilized to validate and implement the ML techniques. The experimental results proved that the proposed ANN model outperforms the SVM and KNN techniques. Research^23^ used KNN and RF techniques to develop a classification model for the HCV dataset of Egyptian patients. This dataset contains two classes, i.e., multi-class and binary classes. The proposed model is implemented using Python and R programming languages.   Author proposed a model using six ML techniques, such as SVM, NN, DT, RF, NB, and BN, to classify and predict HCV. An experimental analysis proved that the RF model performs outstanding over other ML models. In research^24^, ML techniques-based model is presented to detect hepatitis C patients in Egypt’s HCV patient dataset.

Experimental analysis shows that the proposed. The BN method achieved the highest accuracy compared to other existing ML techniques. Research^25^ utilized ML techniques, i.e., SVM, RF, DT, MARS, and BGLM, to implement the automatic diagnosis system to predict HCV. The MARS and BGLM techniques achieve better accuracy % in predicting hepatitis C from the UCI blood samples dataset. Research^26^ presents an unbalanced HCV dataset handling method using SMOTE. An experimental result shows that the proposed model improves the accuracy of HCV prediction results^27^. In research^28^, the authors mainly described the reason and analysis of the “direct-acting antiviral treatment failure” using ML methods. This research utilized records collected from the HCV-TARGET database. This dataset contains the statistics of HCV patients who had to receive an all-oral DAA remedy, and they have positive virologic results. This research utilizes all the social demographic, diagnostic, and virologic statistics in preparation for all the predictive factors (n = 179). Research^29^ used different ML techniques to analyze direct-acting antiviral treatment failure for HCV patients. Table 1 illustrates the related work on predicting the Hepatitis C virus.

**Table 1:** Comparative analysis of existing work on hepatitis C virus prediction.

References	Used techniques	Performance metrics used	Data set and number of instances	Outcomes	Challenges
^45^	RF, SVM, GB	Precision, accuracy, miss rate	Online UCI Dataset, 668 instances	RF achieves 89% precision and a 17.2% miss rate	Cannot predict beyond the range of training data and overfitting issues
^12^	RF, KNN	Precision, accuracy, recall, F-measure, confusion matrix	A laboratory examination dataset with 200 instances	RF achieves 6% better results than KNN and other methods	It performs better for limited data
^13^	SVM, DT, GB, LR, NB, KNN, XGB, RF	Accuracy	Online Kaggle Dataset, with 4462 instances	The DB methods show 91.2 & accuracy over other methods	Fewer data samples utilize
^14^	RF	Precision	UCI data set with 670 instances	RF achieves 89.6% precision over other ML methods	Data inconsistency issues
^15^	RF, SVM	Precision, the miss rate	NCA Hospital dataset with 425 instances	RF achieves 87.6% precision	It performs better for limited data
^16^	SVM, ANN, KNN	Precision, accuracy, recall	Online UCI dataset with 295 instances	ANN Achieves 90.1% precision in training and testing	Limited parameters were considered in the experimental analysis
^17^	SVM, RF, DT, BN, NN, NB	Precision, recall, F-measure, detection rate, and recall	Online Kaggle dataset with 559 Instances	NN performs better and achieves more than 11.6% better results than other methods	It performs better for limited data
^18^	Extreme learning machine	Precision, miss rate	Online Kaggle dataset with 550 instances	Better precision as compared to the SVM method	Limited parameters
^19^	ANN	Accuracy, miss rate	Lahore Hospital dataset with 289 instances	ANN achieves better results in terms of accuracy and miss rate %	Data inconsistency issues
^20^	PSO, GA, REP, DT- C4.5 and CART, ADT, MLR, RT	Precision, recall, accuracy, miss rate	Egypt HCV dataset, with 669 instances	GA methods show better classification outcomes	It performs better for limited data
^21^	SVM, NB, NN, DT	Accuracy and miss rate %	Online UCI dataset with 335 instances	NN achieves better accuracy and misses rate%	Limited parameters were considered in the experimental analysis
^22^	SVM, simulated annealing (SA)	Sensitivity, specificity, precision, and accuracy	Online Kaggle dataset with 295 instances	SVM achieves better results than existing ML methods	Data inconsistency issues
^23^	Binary LR	TPR and accuracy	Online UCI dataset with 269 instances	Binary LR achieves better TPR and accuracy %	Performs better for limited data
^24^	ANN, NN, and SVM	Precision and accuracy	Online UCI dataset with 295 instances	ANN achieves better precision	Limited parameters were used

### Limitation of existing research

Based on the "Existing work" review, we can say there are still some critical challenges in HCV research that need immediate consideration. A few of the key challenges are as follows:

- Poor detection accuracy: many existing strategies in literature accomplished poor accuracy^1,3,5^. It becomes challenging for medical professionals to depend entirely on all these outcomes.
- Utilizes fewer parameters in experimental analysis: some existing research operates limited variables^5,7,11,29^ forecasting fibrosis inside a human liver, which can degrade the model’s performance.
- Utilizes limited data samples: some of the HCV research^2,5,7^ utilizes fewer aspects in their HCV prediction research, which encounters accuracy issues and reduces the system’s performance.

While keeping the above shortcomings of previous techniques in consciousness, we have developed an improved HCV protection model in this research. The main objective of the proposed model is to generate better accuracy and deal with database Imbalancing issues. This research implemented several parameters, i.e., accuracy, precision, f-measure, and recall, to prove the effectiveness of the proposed model. Furthermore, experiments were conducted in various phases on a more extensive set of samples to improve the quality and precision of the proposed ML model.

## Materials and methods

This section covers the working of the proposed HPM model and existing ML methods.

### Proposed HPM model

**Figure 2:**
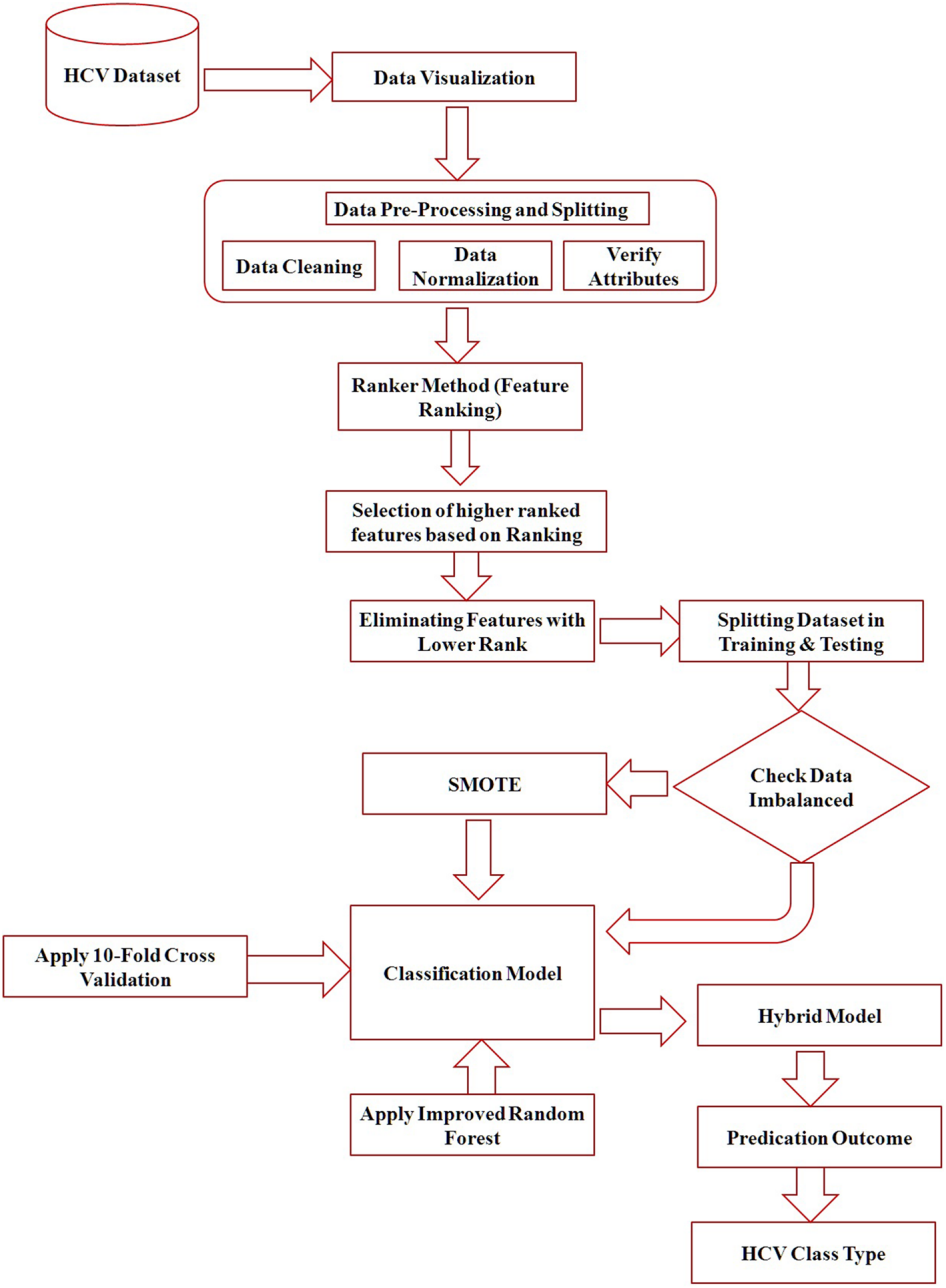
Architecture of proposed hybrid model.

This research proposed a Hybrid Predictive Model for Hepatitis C detection based on IRF and SVM. A new diagnosis system predicts HCV using a data sample with the maximum detection rate in four classes. The effective classification process of blood reports into these classes is crucial for patients suffering from the Hepatitis C virus. Figure 2 shows the implementation architecture of the proposed model. The proposed model determines which features are required for the classification using the feature ranking methods.

A subset of top-ranked attributes is then chosen depending on the ranking. Further, the IRF method is trained using the HCV dataset and generates the best solution through feature selection and removal. The proposed model executes tenfold cross-validation throughout the training phase. Cross-validation is a process for evaluating the performance of the prediction models that divides the samples into training and testing datasets. The initial participants are randomly divided into equal sample groups (10 sub-groups). One subset is kept as validation data to test the classifier. In contrast, the remaining subsets are utilized as training samples in tenfold cross-validation.

#### Improved random forest

RF is a supervised ML technique that builds a forest with many decision trees^27,30^. The main idea behind the RF development is the forest and elections. Each decision tree acts as a voter in the forest. The proposed HPM model improves the RF method by adding bootstrapping method. This IRF method helps to eliminate the less critical features from the tree iteratively to build a strong forest, enhancing the RF model’s performance. The proposed HPM model utilized a Ranker method to rank the dataset features and further applied an IRF with SVM, selecting higher-ranked feature elements to build the prediction model. The IRF employs an optimal count of trees.

**Figure 3:**
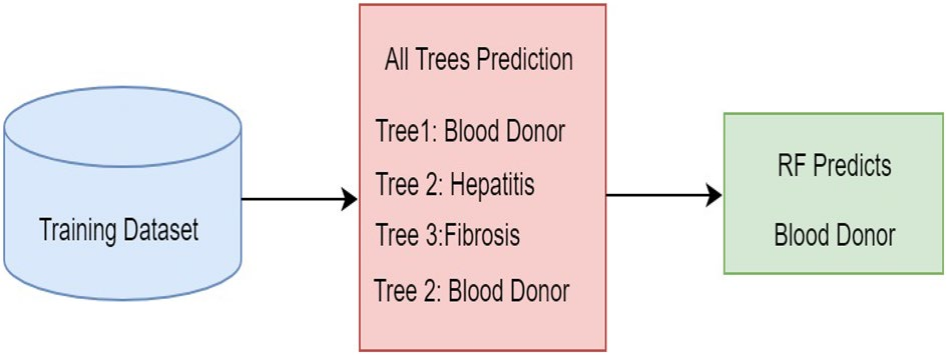
Structure of IRF technique.


In contrast, conventional RF infers that expanding the count of trees dynamically improves the correctness, which is not feasible in practice^31^. IRF also selected the features in a semi-random fashion for splitting. A random subset from the specified data portion is selected from the potential splitting space of features. The prediction accuracy of the proposed system is enhanced by increasing the number of decision trees. RF requires two main input parameters in the construction process: the number of decision trees and attributes at every node. Figure 3 presents the structure of IRF, and the steps are depicted in Algorithm 1.

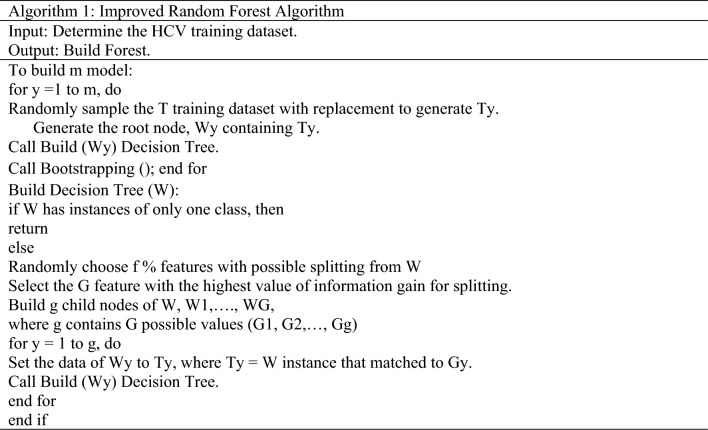


#### Feature ranking process and selection

A Ranker algorithm is used to score and rank the dataset features. The ranker algorithm ranks each feature set in the sample concerning the response variable. The proposed HPM model used a Ranker method to rank the dataset features and further applied an IRF with SVM, selecting higher-ranked feature elements to build the prediction model^32,33^.

#### SMOTE method

A SMOTE is a sampling technique. It randomly creates additional minority class occurrences from the pattern’s minority class neighbors. These individuals are constructed using features from the initial data to complete actual minority class samples. The SMOTE approach is used in the proposed HPM model to resolve data imbalance concerns. SMOTE uses Eq. (1) to create a new minority class^34^.

1 $$\mathrm{Anewf}=(\mathrm{Ai}+\left(\mathrm{AselectedF}-\mathrm{Ai}\right)*\mathrm{t}$$

A SMOTE initially determines the feature set Ai and finds the neighboring elements to verify the data imbalance. It later determines the difference between the new feature set and the old one and multiples it by a random value from 0 to 1. Finally, it adds the outcomes to the feature set to determine a novel data point on a particular line segment. This process is repeated for all the feature sets.

### Existing ML methods

In this study, five ML models, such as SVM, MARS, BGLM, RF, and DT, have been used to develop a Hepatitis C prediction model described below.

#### Support vector machine (SVM)

**Figure 4:**
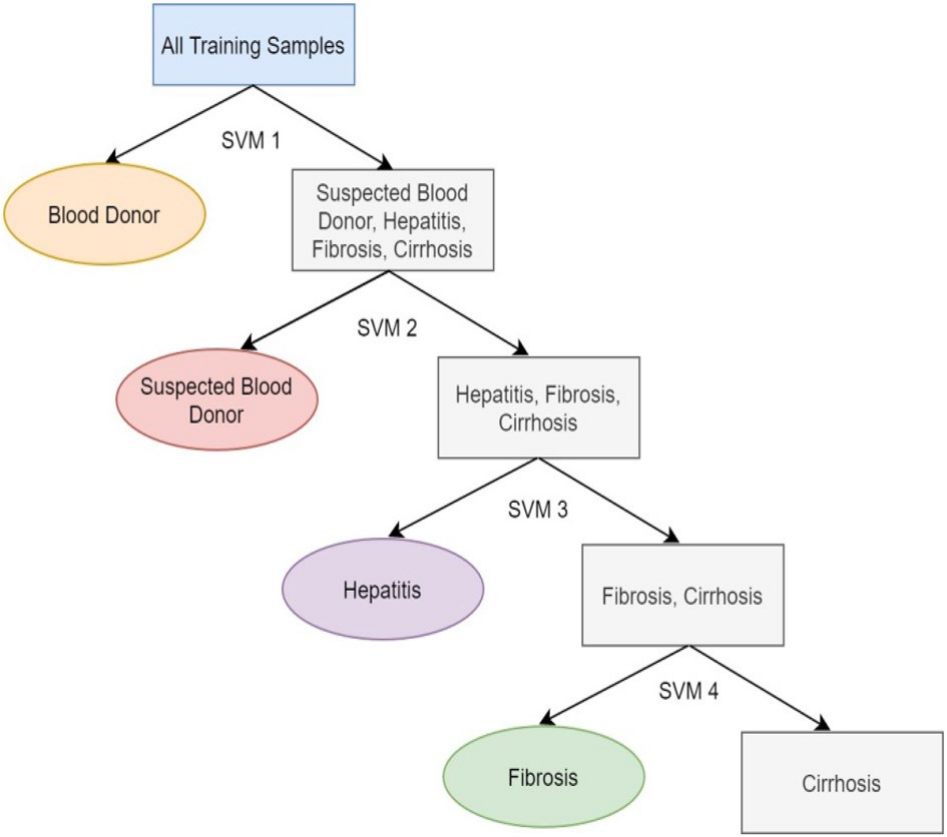
Structure of SVM technique.


SVM is an efficient, popular, and powerful supervised ML technique for prediction problems. It extracts the different data points and segregates them into the n-dimensional feature space, utilizing a non-linear kernel function. In this, hyper-planes are generated using a labelled training HCV dataset for separating the feature space by their severity classes. A new category is assigned to labelled classes utilizing the prediction dataset^35,36^. The SVM technique is described in Algorithm 2 and Fig. 4.

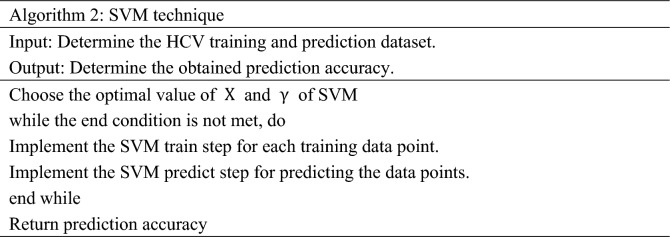


The working of SVM depends on two main steps. Initially, SVM finds the decision boundaries that precisely classify the training HCV dataset. After that, SVM chooses the boundary that has the maximum distance from the nearby data points. The primary aim of SVM is to split the class by searching for the optimal hyperplane^37^. It has some parameters that require tunings, such as x and y. The x parameter governs the interaction between the accurate prediction and smooth decision boundaries of training data points. Suppose the x parameter has a significant value for accurately obtaining more training data points. In that case, a complex curve boundary is generated that fits all the data points. To avoid the overfitting issue and get a perfectly stable curve, different values of x are required for the dataset. The γ parameter is used to describe the single training impact. The high value of the γ parameter indicates that a data point has nearby reachability. In contrast, the low weight of the γ parameter suggests that each data point has a substantial space.

#### Decision tree (DT)

DT is a supervised ML technique used to solve the prediction problem by learning the decision rules^38,39^. In the construction of DT, the process starts from the root node for predicting a class from the input training data. The best attributes are placed at the root of the tree. The input training data is split into subsets, and root attribute values are compared with the data attributes. For comparison, the branch resultant to that value is followed for selecting the next leaf node. The above steps are repeated until a leaf node with a predicted class label is found. The main goal of the tree-building process is the attribute splitting that creates the best possible child nodes. The steps of the DT technique are illustrated in Algorithm 3.
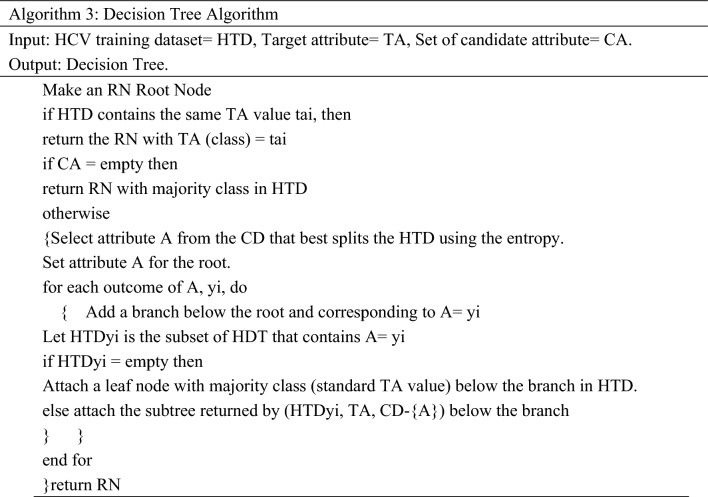


#### Multivariate adaptive regression splines (MARS)

MARS is a non-parametric and non-linear flexible regression technique implemented by Friedman. It provides accurate results for high-dimensional problems with more than one input variable^1^. In this algorithm, predicted and dependent variables have no assumption about their functional relationship. It provides surety in fitting the functions of non-linear multivariate^40^. Therefore, it has been widely utilized for disease prediction in the past few years. MARS required a set of Basis Functions (BF) and coefficients of the given predictor (y) and valued u as presented in Eq. (2).

2 $${\left(\mathrm{y}-\mathrm{u}\right)}_{+}=\left\{ \begin{array}{l}y-uy>u\\ 0, otherwise\\ uy\end{array}\right\}$$

where the + sign defines the positive part. Let us assume y is the patient’s age, the value of the best split (u) is age 54, then (54-y)_+ and (y-54)_+ denote the region that is lower and greater than 54, respectively. The MARS model is presented using Eq. (3).

3 $$\mathrm{x}=\mathrm{f}\left(\mathrm{y}\right)={\mathrm{A}}_{0}+\sum_{\mathrm{t}=1}^{\mathrm{T}}{\mathrm{A}}_{\rm{t}}{\mathrm{H}}_{\rm{kt}}\left({\mathrm{x}}_{\rm{v}(\mathrm{kt})}\right)$$

where x represents the dependent variable, T is the term, A_0, and A_tare the two parameters that are assessed from the HCV training dataset. H is the function that is defined using Eq. (4).

4 $${\mathrm{H}}_{\rm{kt}}\left({\mathrm{x}}_{\rm{v}\left(\mathrm{kt}\right)}\right)=\prod_{\mathrm{k}=1}^{\mathrm{K}}{\mathrm{h}}_{\rm{kt}}$$

where ${\mathrm{x}}_{\rm{v}(\mathrm{kt})}$ acts as the predictor in the kth item. Further, it has three main steps that are described below:

- Forward pass: BF is added in pairs to the model based on the maximum predetermined reduction in the sum of the best square fit.
- Backward pass: the BF of overfitting is removed from the model. For building a good fit model to the data, a Generalized Cross-Validation (GCV) error is calculated, taking the model’s residual error and complexity. It can be represented with the help of Eq. (4).
  5 $$\mathrm{GCV}= \frac{\sum_{\mathrm{i}=1}^{\mathrm{M}}{\left({\mathrm{x}}_{\rm{i}}-\mathrm{f}\left({\mathrm{y}}_{\rm{i}}\right)\right)}^{2}}{{\left(1-\frac{\mathrm{D}}{\mathrm{M}}\right)}^{2}}$$
  6 $$\mathrm{D}=1+\mathrm{de}$$

Where M represents the number of patients in the dataset, d is defined as a freedom degree equal to numerous independent BF, and C describes the penalty for adding BF. The MARS model uses the cross-validation method to predict the optimum results. The model has a higher accuracy rate and lower mean square error.

#### Bayesian generalized linear model

BGLM is a linear regression technique that is used for constructing relationships. It removes the overfitting issue and provides a good fit for the dataset in a pragmatic size^41^. As the name suggests, it takes the prior distribution based on preliminary data. After that, sample information is integrated with the primary data to obtain the posterior distribution. The information provided by posterior distribution is nearer to accurate information since it combines expert opinions and sample information. The “arm” package implements the BGLM in the R programming language.

### Performance measuring parameters

Figures should have relevant legends but should not contain the same information already described in the main text. Figures (diagrams and photographs) should also be numbered consecutively using Arabic numbers^42,43^. They should be placed in the text soon after the point where they are referenced. Figures must be submitted in digital format, with a resolution higher than 300 dpi. This research utilizes the following key parameters to measure the performance of the proposed and existing model^35–37^.

- Accuracy: indicates the correctly predicted blood samples from a blood donor, suspected blood donors, Hepatitis, fibrosis, and Cirrhosis. The accuracy of the proposed system is calculated using Eq. (7).
  7 $$\mathrm{Accuracy}=\frac{\mathrm{PS}+\mathrm{NS}}{\mathrm{PS}+\mathrm{NS}+\mathrm{FS}+\mathrm{IS}}$$

Where PS is the positive samples that are correctly classified, NS is the negative samples that are correctly classified; FS represents the negative samples that are classified as positive samples, and IS denotes the positive samples that are classified as negative samples PS and NS represent the correctly classified samples. In contrast, FS and IS are the incorrectly classified samples.

- Precision: represents the actual negative values that can be correctly classified and calculated using Eq. (8).
  8 $$\mathrm{Precision}=\frac{\mathrm{PS}}{\mathrm{PS}+\mathrm{FS}}$$
- Recall: it indicates actual positive values among all positive ones and can be estimated with the help of Eq. (9).
  9 $$\mathrm{Recall}=\frac{\mathrm{PS}}{\mathrm{PS}+\mathrm{IS }}$$
- F-measure: it can be computed using recall and precision as given in Eq. (10). It is unaffected by negative values.
  10 $$\mathrm{F}\_\mathrm{measure}=\frac{2\mathrm{PS}}{2\mathrm{PS}+\mathrm{PS}+\mathrm{IS}}$$

## Results and discussion

This section covers the experimental detail, dataset description, pre-processing, and results from validation and discussion.

### Data pre-processing

**Figure 5:**
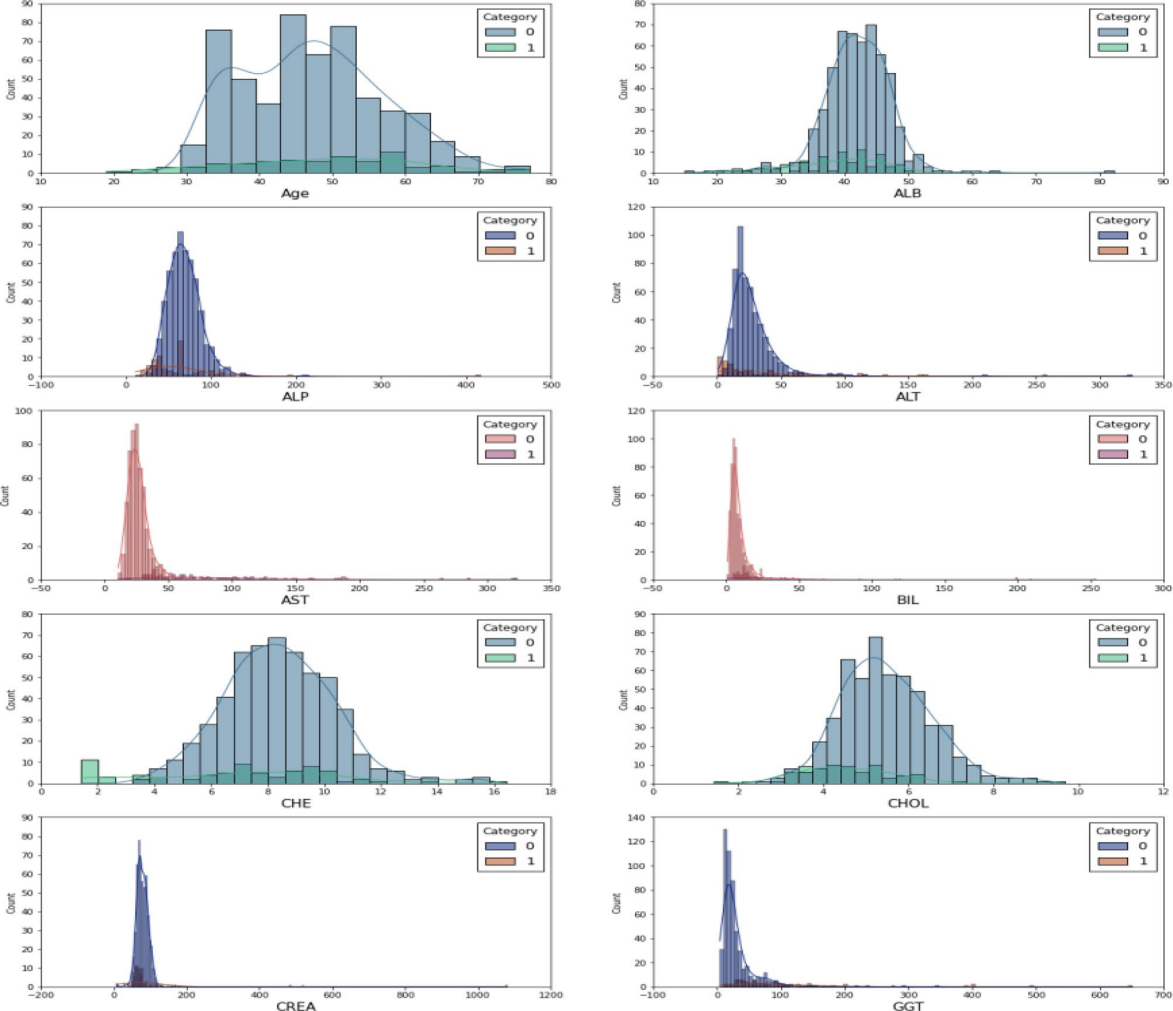
Dataset description histogram with class details (Class 0 and 1)*.*

This research utilizes an online HCV UCI dataset^44^. The UCV HCV dataset contains 1756 records with 29 attributes. In the dataset, 1056 are unhealthy, and 700 records are for healthy people. Table 2 shows the dataset description and class details. Figure 5 shows the details for dataset features with class type. The y label shows to count, and the X label shows a property.

**Table 2:** Dataset description.

Label	Class type	Number of records	Binary class
F1: portal fibrosis	Multi-class	556	Class 0
F2: few septa	Multi-class	500	Class 0
F3: many septa	Multi-class	400	Class 1
F4: cirrhosis	Multi-class	300	Class 1

The missing data produces incorrect predictions and degrades the quality^39^. The primary process in the proposed model is data processing, which includes eliminating noisy data and fixing missing data for particular characteristics. This is presumed that missing, inconsistent, and redundant data statistics have been resolved in the new experimental sample data. As shown in Table 3, most healthcare features were transformed from numeric values to categorical attributes. Therefore, this study does not handle missing data in the HCV dataset. The data augmentation method is utilized to get sufficient testing, training, and validation data. The instances with missing values are removed from the dataset, and an imputation method is applied to the remaining data. The output of this phase is normalized data shown in Table 3.

**Table 3:** Description of dataset features.

S.No	Feature name	Data type	Details	Feature range
1	ID type	Numeric	Patient ID	0 patient ID
2	Age	Numeric	Patient age	[20 to 65]
3	Sex	Binary	Patient sex	0 female and 1 male
4	ALB	Numeric	Albumin quantity in the blood	[14.91 to82.2]
5	ALP	Numeric	Alkaline phosphatase in the blood	[11.31 to 416.6]
6	ALT	Numeric	Aanine aminotransferase (liver damage status)	[0.99 to 325.31]
7	AST	Numeric	Aspartate aminotransferase in liver	[10.61 to 324]
8	BIL	Numeric	bilirubin test value in the blood	[0.81 to 254]
9	CHE	Numeric	Serum cholinesteras (liver function)	[1.421 to 16.41]
10	CHOL	Numeric	cholesterol in blood	[1.431 to 9.671]
11	CREA	Numeric	Creatinine in blood	[8.1 to107.9]
12	GGT	Numeric	Gamma-glutamy transferase (liver disease)	[4.51 to 650]
13	PROT	Numeric	Protein test	[44.81 to 90]

#### Co-relationship and dealing data imbalancing with SMOTE

The correlation with both the result parameter and all actual clinical parameters has been estimated once evolving supervised classifier model. Correlation coefficient matrices describe the correlation classes. The 70% training dataset and the 30% testing dataset were used. The allocation of patient data predicated on the dependent variable demonstrates that the original dataset is imbalanced. Across pre-processing phase, the SMOTE method has been used to tackle this problem^45^.

**Figure 6:**
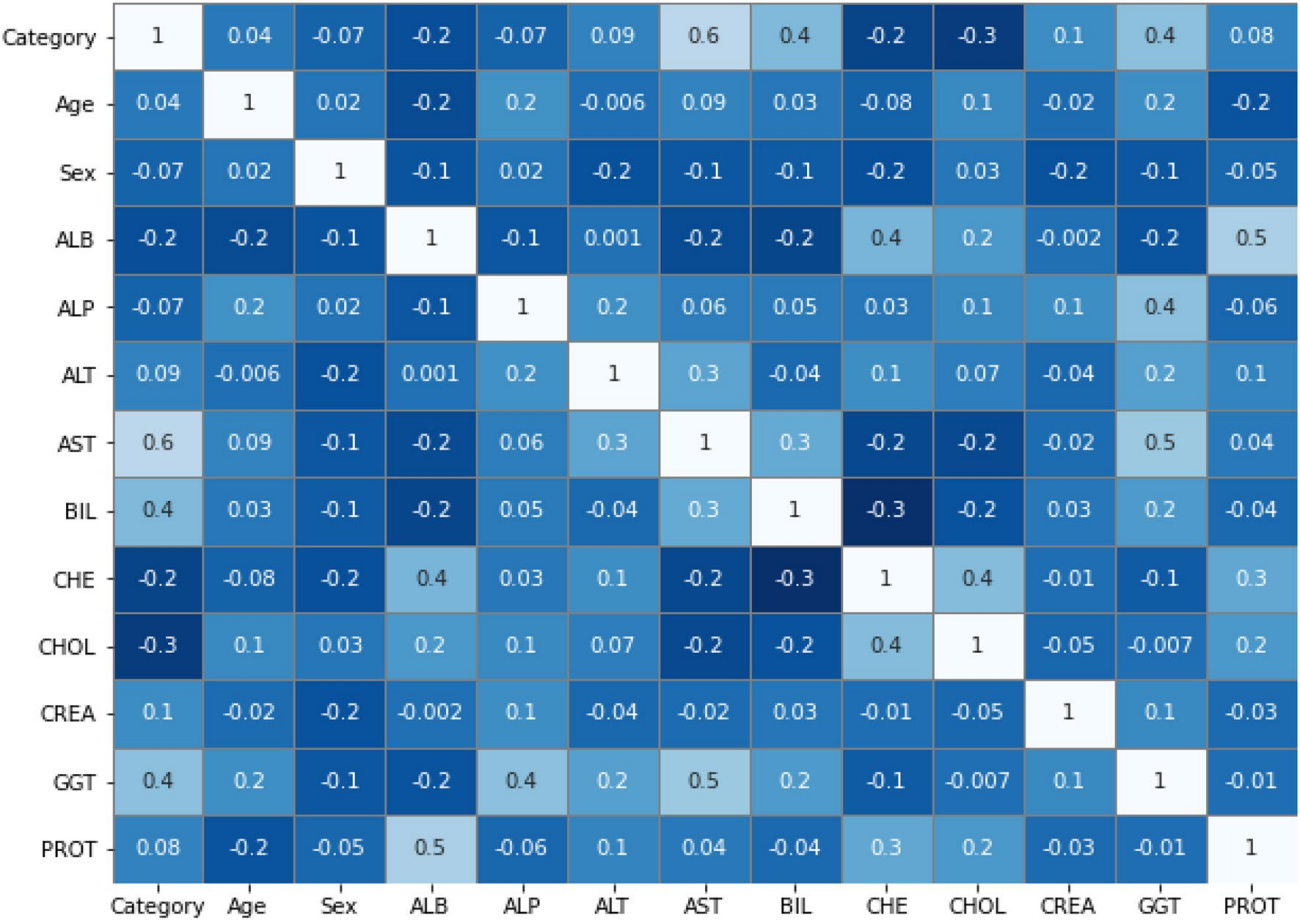
Heat map diagram of correlation of various independent variables in the dataset.

Since utilizing SMOTE, a new data sample again had equivalent volumes of data for outcome measures and was completely ready to be estimated. SMOTE has been implemented on only the training dataset to prevent data leakage and reduce method overfitting. Figure 6 shows the dataset’s heat map of the various independent variables^46^.

#### K-fold method

A K-fold cross-validation method is utilized to split the dataset in training and testing. A cross-validation method is a powerful method in machine learning. The main objective of the cross-validation method is to acquire a stable and consistent estimate of system performance. In a K-fold cross-validation method, the dataset is divided into the k distinct portions. Each iterative process employs k − 1 parts to the training set and the remaining amount to serve as a test dataset. The process is repeated based on the number of folds. The mean of the measured scores signifies the model’s prediction performance. It mainly supports two types of cross-validations, k: 5 and k: tenfold.

### Feature selection using Rankers method

**Figure 7:**
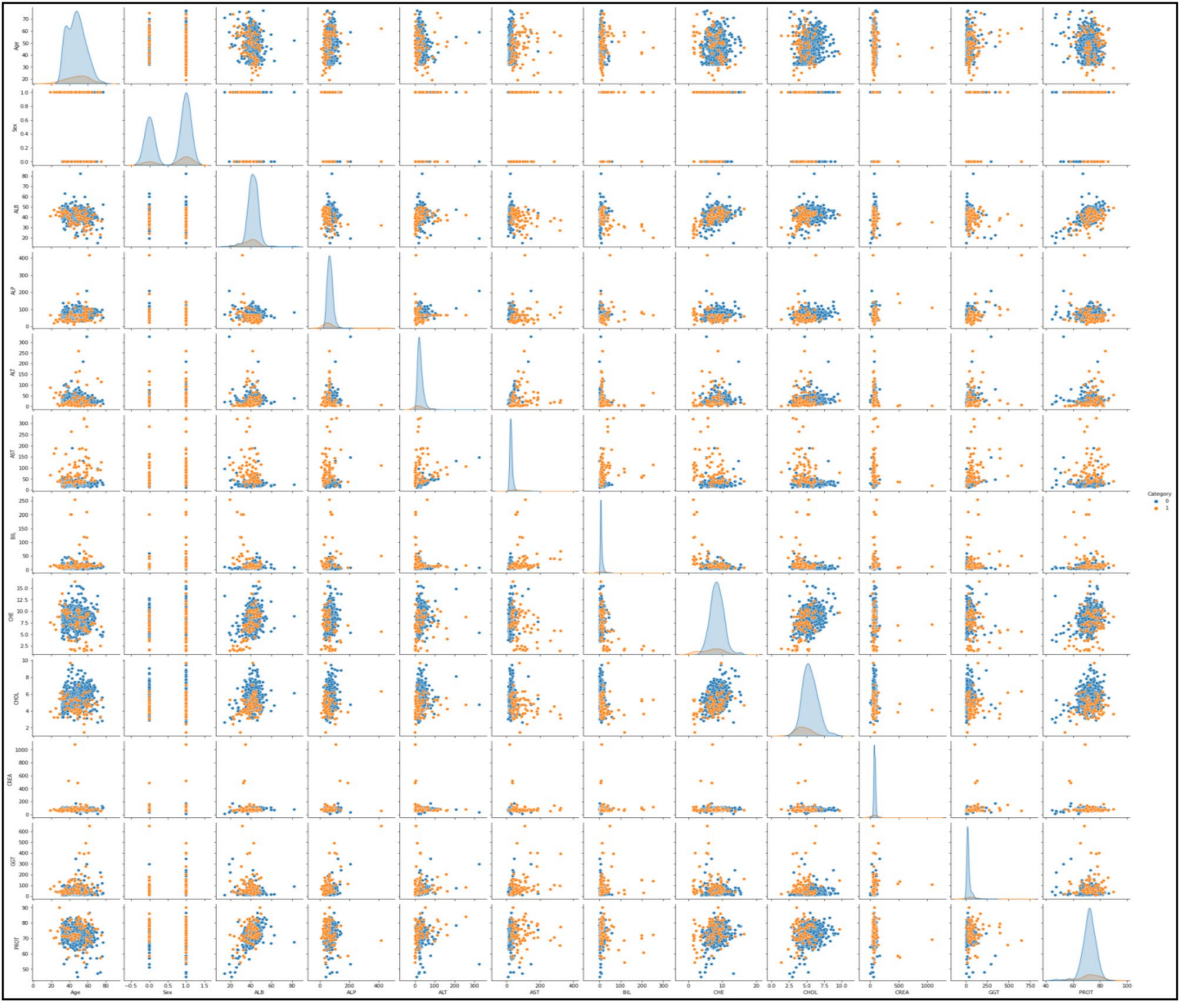
Selected features by the feature selection method.

Feature selection identifies a set of features or factors defining data to generate a much more compact and crucial depiction of the data set while neglecting some other repetitive and unnecessary attributes. Figure 7 shows the selected features after applying the feature selection method. We performed our simulation on a 3.0 GHz (4.7 GHz Turbo) computer with 8 GB RAM and 64-bit Windows OS. The proposed HPM model and existing ML models are implemented using python programming language under the Anaconda environment^38^. The five ML models, SVM, DT, RF, BGLM, and MARS^2,3,5^, are compared with the proposed HPM model.

The proposed system utilized Rankers methods for feature selection. Ranker’s method first uses variable ranking (VR). VR is the procedure of ranking features based on the significance of a scoring function that typically attempts to evaluate feature relevance for all the attributes. Equation (11) shows the correlation calculation function. Higher values show better features. Here R (fi, y) indicates the Correlation Coefficient between feature and target, and cov shows coverage and offers the correlation value.

11 $$\mathrm{R}\left(\mathrm{fi},\mathrm{ y}\right)=\frac{\mathrm{cov}\left(\mathrm{fi},\mathrm{ y}\right)}{\sqrt{\mathrm{var}(\left(\mathrm{fi}\right),\mathrm{ var}(\mathrm{y})}}$$

In the proposed system, the ranker’s method selected 21 features out of 29 features from the HCV dataset. The ranker’s approach considers those parameters that can cause Hepatitis C disease. It calculates the correlation value by Eq. (11). Higher R(fi,y) values were considered in the experiment.

#### Experiment 1 based on data-splitting methods

The effectiveness of ML algorithms depends on the statistics’ quality and the methodology used. Consequently, evaluating the effect of data splitting on ML algorithm outcomes is critical because it will redevelop the path for enhanced ML-based data analysis by enabling an appropriate statistics-splitting strategic approach. We compared acceptable data partitioning methods using real-world HCV datasets and all characteristics. In this research, the dataset was split using the K-fold cross-validation method and the training–testing partition technique.

In experiment 1, the dataset was split into two parts using the random splitting technique, with various ratios: 80:20 and 70:30 (training: testing). In the second phase, the data set was divided into two parts using a k-fold. In the k-fold cross-a validation method, we utilize the parameters k = 5 for the first split and k = 10 for the second split. In the first experiment, we calculated the accuracy of various ML methods. We proposed the HPM method for the UCI HCV dataset for k = 5 for the first split and k = 10 for the second split. Table 4 shows the accuracy results of various methods.

**Table 4:** Experimental results (accuracy %) of five ML techniques using the HCV dataset.

Models	Experiment (based on data splitting method)			
	K-fold cross validation split method		Training:testing, split method	
	K: 5	K: 10	80:20	70:30
SVM	89.88	89.95	90.25	90.45
MARS	87.68	88.56	88.12	87.90
RF	89.95	90.78	90.12	89.41
DT	89.31	89.96	89.69	88.49
BGLM	87.68	88.97	86.99	85.47
Proposed HPM	95.89	96.29	91.24	92.39

### Discussion

The experiment is based on the data splitting method K-fold cross-validation and training: testing based split on normalized HCV dataset. The main motive of experiment 1 is to improve the accuracy of HCV detection. In previous research, the dataset was imbalanced. So firstly, we applied SMOTE with the Rankers method to deal with an imbalanced dataset and select the best features. Now the data set has only relevant features. In this experiment, we are using a total of 21 features out of 22. The highly co-relevant features are selected (discussed in the next section, Fig. 7). Based on experiment results of experiment 1, we can see that when ML classification methods use the k-fold cross-validation method with k = 10, their results are better in most contexts, as shown in Table 4. We can see that utilizing tenfold cross-validation well with the proposed HPM method achieves the best results. Consequently, through this research, the tenfold cross-validation technique for dividing the HCV samples is first proven to be the dominant choice for ML modeling techniques. tenfold cross-validation performs the fitting method ten times and generates the best results for the limited dataset.

#### Experiment 2 based on feature selection

**Figure 8:**
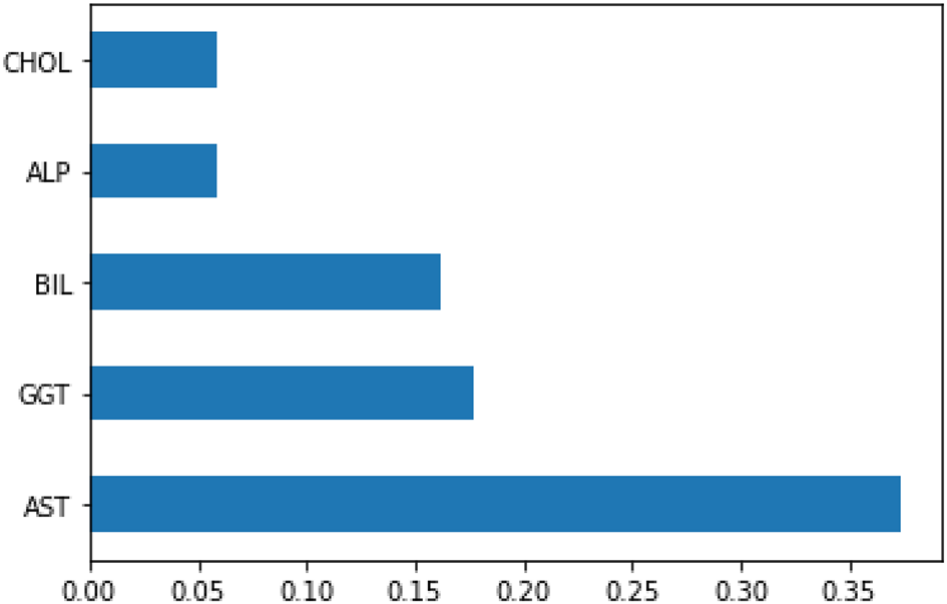
Experimental results for feature selection.

**Figure 9:**
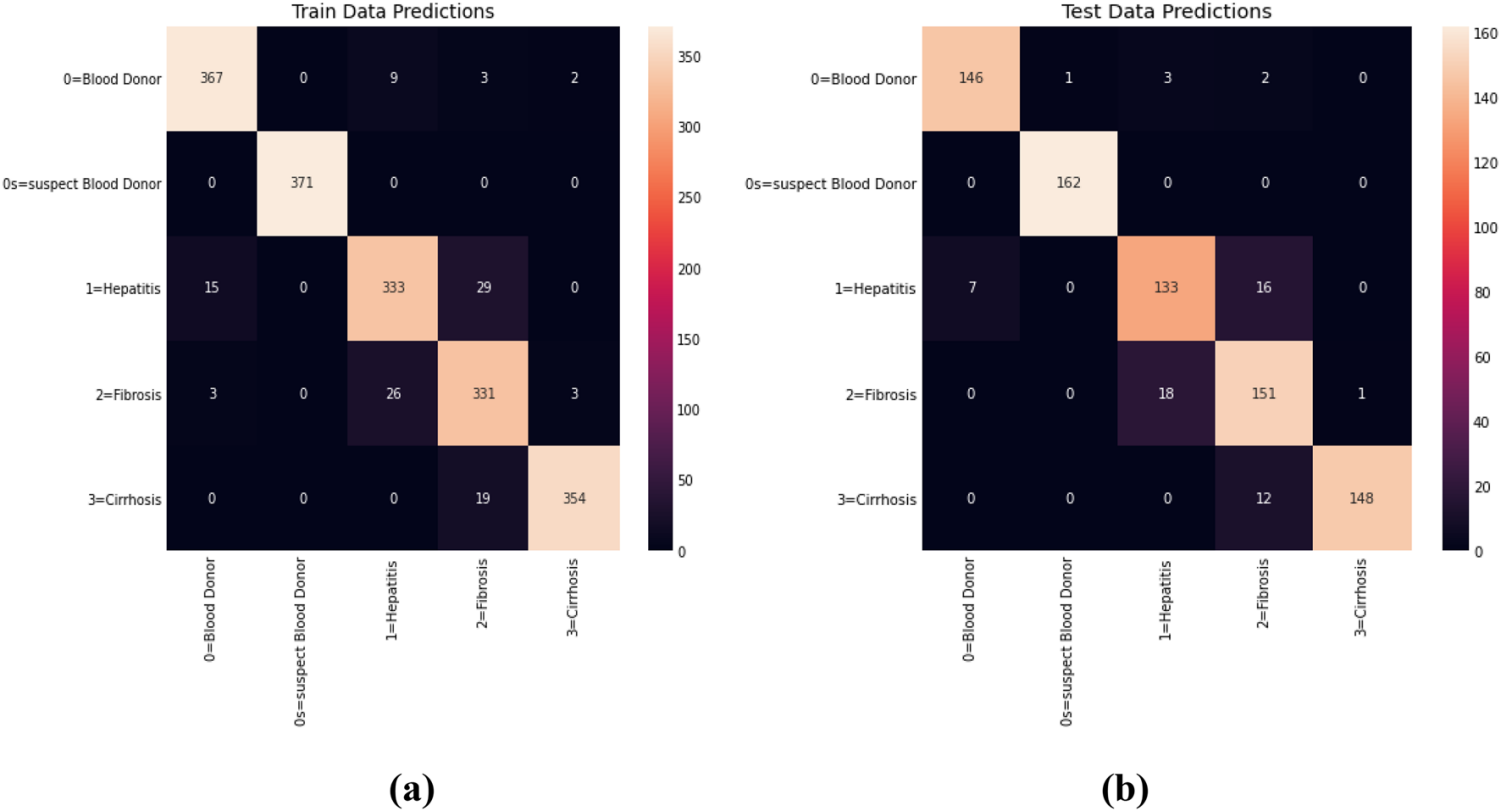
**(a)** Training dataset prediction and (**b**) testing dataset prediction of experiment 2.

In experiment 2, an analysis is performed on selected features. SMOTE method is applied to the dataset to determine the essential features. Figure 8 shows the feature selection method results. This graph offers an attribute’s highly correlated feature results (in %). Figure 9 shows the training and testing dataset prediction for experiment 2. Experiment 2 results are calculated in two-phase first without SMOTE and second with SMOTE method on the HCV dataset.

Table 5 shows an experimental result on the HCV dataset without applying the SMOTE method. Table 6 shows experimental results with the SMOTE method of existing ML methods and proposed HPM methods.

**Table 5:** Experimental results without SMOTE method HCV dataset.

Models	Performance metrics (in %)			
	Precision	Recall	F-measure	Accuracy
SVM	35.682	31.256	35.897	33.471
MARS	33.471	32.562	32.442	31.684
RF	38.745	36.554	37.998	37.521
DT	35.336	35.102	34.787	35.447
BGLM	30.747	31.245	30.451	32.232
Proposed HPM	41.223	40.556	42.332	41.541

**Table 6:** Experimental results with SMOTE method HCV dataset.

Models	Performance metrics (in %)			
	Precision	Recall	F-measure	Accuracy
SVM	94.50	96.67	95.01	96.61
MARS	93.88	94.65	94.26	96.05
RF	89.90	96.78	93.21	95.48
DT	96.09	93.67	94.86	94.35
BGLM	90.00	91.22	90.61	93.22
Proposed HPM	98.93	99.13	97.54	96.82

## Discussion

**Figure 10:**
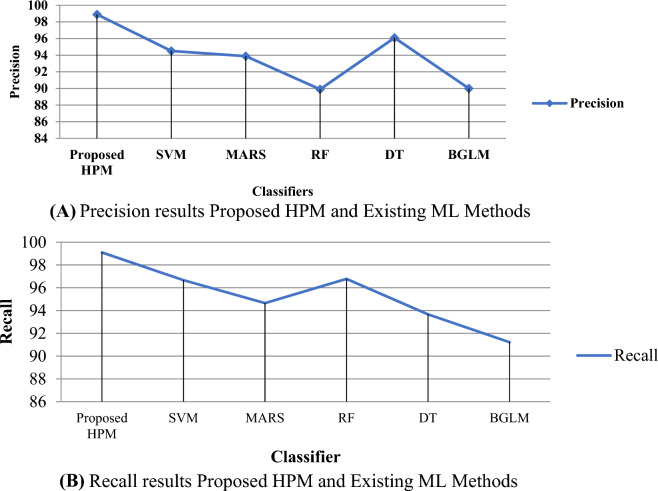
**(A)** Precision results proposed HPM and existing ML methods. **(B)** Recall results proposed HPM and existing ML methods.

**Figure 11:**
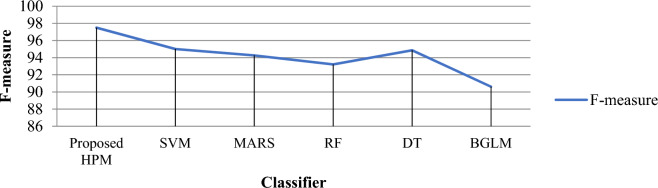
F-measure results proposed HPM and existing ML methods.

**Figure 12:**
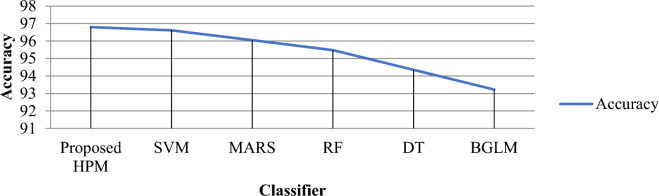
Accuracy results proposed HPM and existing ML methods.

Tables 5, 6, and Figs. 9, 10, 11 and 12 demonstrate the experimental results of the proposed Hybrid Predictive Model (HPM) and existing ML techniques using HCV datasets without SMOTE and with SMOTE. In the first phase, when we utilize the HCV dataset without SMOTE method (Table 5), the proposed method achieves a precision of 41.23% and accuracy of 41.541%, Recall of 40.556%, and F-measure 42.332%, which are the highest as compared to existing ML methods. In the second phase (Table 6), an experimental analysis is performed on the HCV dataset by applying SMOTE method. The proposed model achieved higher precision, Recall, F-measure, and accuracy of 98.9%, 99.1%, 97.5%, and 96.8%, which is far better than other existing ML methods. The proposed HPM model utilized a Ranker method to rank the dataset features and further applied an IRF with SVM, selecting higher-ranked feature elements to build the prediction model, which improves the overall performance of the proposed model.

It is observed that the SVM model achieved precision, Recall, F-measure, and accuracy of 94.50%, 96.67%, 95.01%, and 96.61%, respectively. The MARS model achieved an accuracy of 96.05% than RF, DT, and BGLM. However, the model outperforms the DT and BGLM models with an F-measure of 93.21% and a success rate of 95.48%. The DT model achieves better prediction results than BGLM in all performance metrics. The precision, Recall, F-measure, and accuracy of the DT model are 96.09%, 93.67%, 94.86%, and 94.35%, respectively. It is noticed that BGLM based hepatics C prediction model exhibits the worst result among all the prediction models by obtaining 93.22% accuracy.

## Conclusion and future works

Early and accurate detection of Hepatitis is always in demand. The ML-based model plays a vital role in health care research, i.e., disease detection, classification, level protection, and correct diagnostics. The ML models suggested by earlier research encounter several issues, i.e., poor accuracy, missing values, irrelevant feature selection, and poor performance. This research developed a Hybrid Predictive Model “HPM” to deal with these above-discussed issues. The proposed model utilizes a Ranker method for feature selection from the HCV dataset. The ranker method selects only highly correlated features and eliminates irrelevant features. The proposed model uses a Ranker method for feature selection from the HCV dataset. The ranker method determines only highly correlated features and eliminates irrelevant features. It helps to improve the accuracy of the model.

This research conducted two experiments to measure the performance of the proposed model and the existing ML model (discussed in earlier research). The main motive of the study is to enhance HCV detection accuracy. In experiment 1, two data-splitting techniques are used. The first technique is based on k-fold cross-validation, and the second is based on training testing split. The second experiment is based on the feature selection process from the HCV dataset. It includes two types of analysis, one with SMOTE and another without SMOTE. The proposed HPM model is compared with well-known ML methods utilized to be earlier researchers in HCV detection. Experimental analysis shows that in experiment 1, for K-fold cross-validation, the proposed method achieved an accuracy of 95.89% for k = 5 and 96.29% for k = 10. For the second method of training: testing-based split, the proposed method gained 91.24% for 80:20 and 92.39% for 70:30, which is the best compared to SVM, MARS, RF, DT, and BGLM methods. The proposed method not only improves the detection accuracy but also handles the data Imbalancing issues.

The limitation of the proposed model is its database dependency. The accuracy of the model depends on the quality of the training model. Existing available HCV datasets are static. To mitigate this issue in future work, we will add an IoT-based model to collect real-time statistics on HCV patients. It will help to improve the database quality and prediction accuracy. We will also try to develop more ensembles and a hybrid ML-based model to predict the HCV risk on a real-time dataset.

## Data Availability

The datasets used and/or analysed during the current study available from the corresponding author on reasonable request.

## Abbreviations

ALB: Albumin blood test
ALP: Alkaline phosphates
ALT: Alanine transaminase
ANN: Artificial neural network
AST: Aspartate transaminase
BIL: Bilirubin
BN: Bayesian network
CHE: Acetylcholinesterase
CHOL: Cholesterol
CREA: Creatinine
GGT: Gamma-glutamyl transferase
HCV: Hepatitis C virus
HPM: Hybrid Predictive model
IRF: Improved random forest
DT: Decision tree
BGLM: Bayesian generalized linear model
MARS: Multivariate adaptive regression splines
PROT: Proteins
SVM: Support vector machine
SMOTE: Synthetic minority over-sampling technique
MLP: Multilayer perceptron
KNN: K-nearest neighbor
RT: Regression tree
MLR: Multi-liner regression
ADT: Alternative DT
GA: Genetic algorithm
REP-Tree: Reduced error pruning tree
PSO: Particle swarm optimization
LR: Logistic regression
NB: Naive Bayes
